# Pathways to diagnosis of endometrial and ovarian cancer in the 45 and Up Study cohort

**DOI:** 10.1007/s10552-022-01634-2

**Published:** 2022-10-09

**Authors:** Sarsha Yap, Amy Vassallo, David Goldsbury, Dianne L. O’Connell, Alison Brand, Jon Emery, Anna DeFazio, Karen Canfell, Julia Steinberg

**Affiliations:** 1 The Daffodil Centre, The University of Sydney, a joint venture with Cancer Council NSW, 153 Dowling St, Woolloomooloo, Sydney, NSW 2011 Australia; 2 Cancer Council NSW, Sydney, NSW Australia; 3 The George Institute for Global Health, University of New South Wales, Sydney, NSW Australia; 4 Sydney School of Public Health, Faculty of Medicine and Health, University of Sydney, Sydney, NSW Australia; 5 School of Medicine and Public Health, University of Newcastle, Newcastle, NSW Australia; 6 Department of Gynaecological Oncology, Westmead Hospital, Sydney, NSW Australia; 7 Westmead Clinical School, University of Sydney, Sydney, NSW Australia; 8 Centre for Cancer Research and Department of General Practice, Faculty of Medicine, Dentistry and Health Sciences, The University of Melbourne, Melbourne, Australia; 9 School of Medical Sciences, Faculty of Medicine and Health, University of Sydney, Sydney, NSW Australia; 10 Centre for Cancer Research, The Westmead Institute for Medical Research, Sydney, NSW Australia

**Keywords:** Endometrial cancer, Ovarian cancer, 45 and Up Study, Pathways to diagnosis, Healthcare utilization, Australia

## Abstract

**Purpose:**

To determine pathways to endometrial or ovarian cancer diagnosis by comparing health service utilization between cancer cases and matched cancer-free controls, using linked health records.

**Methods:**

From cancer registry records, we identified 238 incident endometrial and 167 ovarian cancer cases diagnosed during 2006–2013 in the Australian 45 and Up Study cohort (142,973 female participants). Each case was matched to four cancer-free controls on birthdate, sex, place of residence, smoking status, and body mass index. The use of relevant health services during the 13–18-, 7–12-, 0–6-, and 0–1-months pre-diagnosis for cases and the corresponding dates for their matched controls was determined through linkage with subsidized medical services and hospital records.

**Results:**

Healthcare utilization diverged between women with cancer and controls in the 0–6-months, particularly 0–1 months, pre-diagnosis. In the 0–1 months, 74.8% of endometrial and 50.3% of ovarian cases visited a gynecologist/gynecological oncologist, 11.3% and 59.3% had a CA125 test, 5.5% and 48.5% an abdominal pelvic CT scan, and 34.5% and 30.5% a transvaginal pelvic ultrasound, respectively (versus ≤ 1% of matched controls). Moreover, 25.1% of ovarian cancer cases visited an emergency department in the 0–1-months pre-diagnosis (versus 1.3% of matched controls), and GP visits were significantly more common for cases than controls in this period.

**Conclusion:**

Most women with endometrial or ovarian cancer accessed recommended specialists and tests in the 0–1-months pre-diagnosis, but a high proportion of women with ovarian cancer visited an emergency department. This reinforces the importance of timely specialist referral.

**Supplementary Information:**

The online version contains supplementary material available at 10.1007/s10552-022-01634-2.

## Introduction

Gynecological cancers represent almost 15% of new cancer diagnoses and 12% of cancer deaths for women globally, and 10% of cancer diagnoses for women in Australia [1, 2]. Endometrial and ovarian cancers are the two most common gynecological cancers in Australia. Neither have an effective screening test for asymptomatic women [3–5] and detection primarily relies on prompt symptom recognition by women and healthcare professionals. However, other features of disease etiology and epidemiology are markedly different. Approximately 3,000 Australian women are diagnosed with endometrial cancer annually, the symptoms are more specifically gynecological in nature (such as abnormal vaginal bleeding), it is typically detected at an early stage, and the 5-year survival is 82%. By contrast, ovarian cancer is less common with approximately 1,600 diagnoses annually, and the symptoms are non-specific and primarily abdominal or gastrointestinal in nature (such as abdominal pain and bloating) [6]. Early diagnosis is uncommon, with 75% of women diagnosed with advanced stage ovarian cancer [7], contributing to overall 5-year survival of 46% [8]. For both cancers, timely diagnosis and treatment are important for optimal health service delivery and patient care. Understanding the current pathways to diagnosis may allow suboptimal pathways and points of inefficiency to be identified, highlighting key areas for future research and contributing to future recommendations and health service practices to improve outcomes for people with cancer.

While rapid proliferation and exfoliation leading to extensive intraperitoneal dissemination contributes to the advanced stage at diagnosis for many ovarian cancers [9], delayed diagnosis may also be partially responsible, due to non-specific symptoms that do not immediately suggest gynecological disease [10]. Data from two Australian studies (using retrospective patient self-reported information) found that 14.7% of women visited ≥ 4 different doctors between the first presentation to a medical professional and clinical diagnosis, 23.2% had ≥ 5 consultations before a diagnosis was made and 11.7% saw a hospital or emergency doctor prior to diagnosis [11]. Despite this, ~ 80% of women were diagnosed with cancer within 3 months of initial health service presentation and 95% received first treatment within 2 months of diagnosis [11, 12]. A large survey of North American women with ovarian cancer found that 64% believed there were health system barriers to a prompt diagnosis [13], including referral to gastroenterologists, a lack of further investigations, and misattribution or non-recognition of symptoms [14]. Given that disease stage at diagnosis is a critical prognostic factor [15], prompt and efficient diagnosis are a critical issue warranting further inquiry [16]. There is some evidence of poor alignment between patients’ recall and medical records regarding time to ovarian cancer diagnosis [17], which suggests investigations using medical records are needed to obtain a comprehensive picture of pathways to ovarian cancer diagnosis.

This study leverages extensive linked data including medical records for the 45 and Up Study cohort to improve understanding of the current pathways to diagnosis. In particular, we assessed healthcare utilization during the 18 months prior to endometrial or ovarian cancer diagnosis. To differentiate patterns of healthcare utilization related to the cancer diagnosis, we compared healthcare utilization between endometrial and ovarian cancer cases and matched controls with no history of cancer.

## Methods

### Study sample

We utilized data from the Sax Institute’s 45 and Up Study, a cohort of 267,153 residents in New South Wales (NSW), Australia, aged ≥ 45 years at recruitment in 2006–2009 (142,973 women). Participants were randomly sampled from the NSW general population using the Services Australia (formerly the Department of Human Services and Medicare Australia) database, which has near-complete coverage of the population. Residents of remote areas and individuals aged ≥ 80 years were oversampled. Overall, 18% of those invited participated, and the cohort represents ~ 11% of the NSW population aged ≥ 45 years. Participants completed a baseline questionnaire covering health and lifestyle information and provided written consent for linkage to their health-related records [18].

### Linkage to population-wide health databases

Baseline questionnaire data were linked to several health databases to identify incident cancers, deaths, and quantify heath service utilization: (1) NSW Cancer Registry (NSWCR; Jan 1994–Dec 2013) containing all NSW cancer notifications to ascertain incident ovarian and endometrial cancers and clinical characteristics; (2) Australian Coordinating Registry Cause of Death Unit Record File (Feb 2006–Dec 2015) and Registry of Births, Deaths, and Marriages (Feb 2006–Dec 2015) to estimate survival after diagnosis; (3) Admitted Patient Data Collection (APDC; Jul 2005–Dec 2013) to determine hospital admissions and pre-existing comorbidities; (4) Emergency Department Data Collection (EDDC; Jan 2005–Dec 2013) to identify emergency department presentations; and (5) Medicare Benefits Schedule (MBS; Jun 2004–Dec 2013) to quantify other healthcare utilization leading up to the cancer diagnosis. MBS captures services provided out of hospital, including out-patient clinics in public hospitals and to private patients admitted to public or private hospitals. Importantly, MBS data do not include services provided to admitted public patients in public hospitals [19]. Participants’ identifiers were probabilistically linked to databases (1)–(4) by the Center for Health Record Linkage (CHeReL, www.cherel.org.au) using a best practice approach to linkage while preserving privacy [20], with data for (1)–(4) provided by the Cancer Institute NSW and the NSW Ministry of Health. For (5), data were supplied by Services Australia and linked by the Sax Institute using a unique identifier.

### Eligibility criteria for incident cases and controls

We identified women in the 45 and Up Study with a NSWCR record of ovarian cancer (including fallopian tube and primary peritoneal cancer) (International Classification of Disease 10th edition, Australian Modification: C48.1, C48.2, C56, C57.0, C57.8) or endometrial (C54.1) cancer diagnosed after recruitment. Ovarian cancers with unspecified or non-epithelial histology (ICD-O-3-morphology code: 8000, 8001, 9580) were excluded. Cases with another NSWCR record prior to the incident cancer were excluded. Women without any NSWCR record were considered potential controls. Exclusion criteria for both cases and controls were self-reported cancer at recruitment (excluding non-melanoma skin cancers), record linkage errors, Department of Veterans’ Affairs (DVA) clients (as information on their health services utilization is incompletely captured by the available linked data), or missing data for any of the matching characteristics. We also carried out a sensitivity analysis without exclusion of DVA clients.

### Matching of cases to controls

To better distinguish patterns of healthcare utilization related to the cancer diagnosis, cases were matched to controls based on self-reported characteristics at recruitment that affect healthcare utilization: date of birth (± 1 year), tobacco smoking (current, quit ≤ 15-years pre-baseline, quit > 15-years pre-baseline, never), Body Mass Index (BMI (kg/m^2^): underweight (< 18.5), normal (18.5 to < 25), overweight (25 to < 30), and obese (≥ 30), and place of residence (according to the Accessibility/Remoteness Index for Australia: major cities, inner regional, and outer regional/remote/very remote [21]). Four controls were matched to each case [22], with matching by date of birth relaxed to ± 2 years to identify four matched controls for a few cases (*n* < 5).

### Participants’ characteristics

For cancer cases, we examined age and spread of disease at diagnosis, tumor histology, and method of diagnosis. For cases and controls, we also considered age at recruitment, month and year of recruitment, country of birth, education, private health insurance, marital status, self-reported menopausal status, derived menopausal status that accounted for self-reported interventions that can mask menopause (including Menopausal Hormone Therapy (MHT) use, oophorectomy and hysterectomy [23]), hormonal contraceptive use, MHT use, family history of each of breast/ovarian/bowel cancers, K10 distress scale [24], and Charlson’s comorbidity index score [25]. Comorbidities were identified from diagnosis codes in hospital records from 5-years pre- to 6-months post-diagnosis.

### Healthcare utilization

To determine healthcare utilization prior to each case’s diagnosis, we considered visits to General Practitioners (GPs), gynecologists and gynecological oncologists (the latter two were considered together in this study as they cannot be distinguished in the MBS data), medical oncologists, gastroenterologists, emergency departments, and CA125 tests, gastroscopies, colonoscopies, abdominal Computed Tomography (CT) scans (including upper abdomen only, and upper abdominal and pelvis), and transvaginal pelvic ultrasounds (TVUSs), using the health data items shown in Supplementary Table 1.

For each case and their matched controls, we considered healthcare utilization records in the 13–18, 7–12, and 0–6 months prior to the case’s diagnosis date, defined in the NSWCR as the earliest definitive diagnostic or treatment episode, with an additional analysis covering 0–1-months pre-diagnosis. As a sensitivity analysis, we excluded the day of diagnosis from the 0–6 and 0–1 months. Further, we conducted a post hoc analysis of healthcare utilization for each month in the 6 months prior to the cases’ diagnoses.

### Statistical analyses

All analyses were conducted using SAS 9.4 or Stata 16. We calculated overall and cancer-specific 1-year and 2-year survival after diagnosis using the Kaplan–Meier method, censoring cases who were alive at the end of the follow-up period (Dec 2015). For cancer-specific survival, those who died from other causes were also censored at their date of death. Differences in characteristics of cases and matched controls were tested using Fisher’s exact test.

For each health service, differences in utilization between cases and controls were tested using Fisher’s exact test. Significance was defined using Bonferroni adjustment for 10 tests (i.e., *p* < 0.005, accounting for the 10 types of health services). To compare the specialist visits with recommendations from the optimal care pathways [5, 26], we also calculated the percentage of ovarian and endometrial cancer cases who in the 6-months pre-diagnosis had seen either a gynecologist/gynecological oncologist, or a medical oncologist, or both types of specialist. As the proportion of ovarian cancer cases who did not have an MBS consultation record with these specialists pre-diagnosis was high (43.1%), we also calculated the proportion who had a hospital admission and/or visited an emergency department in the 0–1-months pre-diagnosis as consultations with these specialists for hospital in-patients are not captured in the MBS data.

We tested for differences in the number of GP visits between cases and their matched controls in each period using the Wilcoxon–Mann–Whitney test.

In a sensitivity analysis, we retained DVA clients as cases and potential controls. We repeated both the matching process and subsequent comparison of healthcare utilization between cases and controls for the 0–6 and the 0–1 months prior to the case’s diagnosis date.

## Results

Of 142,973 female 45 and Up Study participants, we included 238 endometrial cancer cases and 952 matched controls and 167 ovarian cancer cases and 668 matched controls (Supplementary Fig. 1).

### Clinical characteristics

The median age at diagnosis for endometrial and ovarian cancer cases (65 and 68 years, respectively) was similar to that in Australia for uterine cancers, but higher for ovarian cancer (both 63 years) [27].

Of the endometrial cancer cases, 61.3% and 10.1% were diagnosed with local and distant spread of disease, respectively (Table 1). Overall 1- and 2-year survival for endometrial cancer cases were 95.8% and 92.9%, respectively, and cancer-specific survival was 99.1% and 96.5%, respectively.

**Table 1:** Clinical characteristics of endometrial and ovarian cancer cases diagnosed 2006–2013 in the 45 and Up Study, New South Wales, Australia

Characteristics	Endometrial cases	Ovarian cases
	*n* = 238	*n* = 167
	*n* (col %)	*n* (col %)
Spread of disease at diagnosis		
Local	146 (61.3)	16 (9.6)
Regional	54 (22.7)	26 (15.6)
Distant	24 (10.1)	117 (70.1)
Unknown	14 (5.9)	8 (4.8)
Ovarian cancer histology		
Serous		112 (67.1)
Non-serous		40 (24.0)
Unspecified		15 (9.0)
Endometrial cancer histology		
Endometrioid	188 (79.0)	
Other	50 (21.0)	
Unspecified		
Method of diagnosis		
Clinical/imaging/biochemistry	*	7 (4.2)
Histopathology	~ 238 (100.0)^a^	123 (73.7)
Other	*	37 (22.2)

*Numbers < 5 are suppressed to preserve confidentiality

^a^Rounded to 100% due to suppression of small numbers in other categories

Of the ovarian cancer cases, 9.6% and 70.1% were diagnosed with local and distant spread of disease, respectively (Table 1). Overall 1- and 2-year survival for ovarian cancer cases were 86.2% and 72.5%, respectively, and cancer-specific survival was 87.9% and 75.6%, respectively.

### Participants’ characteristics

By design, matching of cases and controls ensured similar distributions for age, place of residence, smoking status, and BMI (Table 2). Cancer cases were similar to matched controls for several other characteristics, including recruitment date and age at recruitment, country of birth, private health insurance, comorbidity score, education, and marital status (Table 2, Supplementary Table 2). A higher proportion of women with endometrial cancer were peri-menopausal compared with their matched controls (+ 7.6%, *p* < 0.001; Table 2), while ever using hormonal contraceptives was less common among ovarian cancer cases than controls (− 12.0%, *p* = 0.015; Table 2).

**Table 2:** Characteristics of endometrial and ovarian cancer cases and matched controls in the 45 and Up Study, New South Wales, Australia

Characteristics	Endometrial cancer			Ovarian cancer		
	Cases	Controls	Fishers exact test	Cases	Controls	Fishers exact test
	*n* = 238	*n* = 952		*n* = 167	*n* = 668	
	*n* (col %)	*n* (col %)	*p*-value	*n* (col %)	*n* (col %)	*p*-value
Age at recruitment, median (IQR)	65 (58–71)	65 (58–71)		68 (59–75)	68 (59–75)	
Recruitment date (m/yy), median (IQR)	7/08 (2/08–9/08)	7/08 (2/08–9/08)		8/08 (2/08–9/08)	7/08 (2/08–9/08)	
Age at diagnosis^a^						
< 65 years	113 (47.5)	455 (47.8)		66 (39.5)	265 (39.7)	
≥ 65 years	125 (52.5)	497 (52.2)		101 (60.5)	403 (60.3)	
Place of residence (ARIA)^b^						
Major	120 (50.4)	480 (50.4)		84 (50.3)	336 (50.3)	
Inner regional	90 (37.8)	360 (37.8)		59 (30.3)	236 (35.3)	
Outer regional/remote/very remote	28 (11.8)	112 (11.8)		24 (14.4)	96 (14.4)	
Cigarette smoking^b,c^						
Never/ex-smoker (quit > 15 years)	206 (86.6)	828 (87.0)		138 (82.6)	560 (83.8)	
Ex-smoker (quit ≤ 15 years)	21 (8.8)	80 (8.4)		20 (12.0)	72 (10.8)	
Current smoker	11 (4.6)	44 (4.6)		9 (5.4)	36 (5.4)	
Body Mass Index^b,c^						
Underweight/Normal (< 25 kg/m^2^)	62 (26.1)	248 (26.1)		66 (39.5)	264 (39.5)	
Overweight (25 to < 30 kg/m^2^)	72 (30.2)	288 (30.2)		55 (32.9)	220 (32.9)	
Obese (≥ 30 kg/m^2^)	104 (43.7)	416 (43.7)		46 (27.5)	184 (27.5)	
Country of birth						
Australia	190 (79.8)	725 (76.2)	0.488	124 (74.3)	512 (76.6)	0.585
Not Australia	47 (19.7)	221 (23.2)		42 (25.1)	148 (22.2)	
Missing	1 (0.4)	6 (0.6)		1 (0.6)	8 (1.2)	
Private health insurance						
Yes	131 (55.0)	590 (62.0)	0.138	97 (58.1)	407 (60.9)	0.707
No	101 (42.4)	340 (35.7)		66 (39.5)	248 (37.1)	
Missing	6 (2.5)	22 (2.3)		4 (2.4)	13 (1.9)	
Menopausal status (consolidated derived)^d^						
Pre-menopause	11 (4.6)	71 (7.5)	< 0.001	8 (4.8)	43 (6.4)	0.508
Post-menopause	195 (81.9)	779 (81.8)		147 (88.0)	557 (83.4)	
Peri-menopause	30 (12.6)	48 (5.0)		*	31 (4.6)	
Unknown	2 (0.8)	54 (5.7)		*	37 (5.5)	
Age at menopause, median (IQR)	52 (50–54)	50 (47–53)		50 (48–52)	50 (45–52)	
Hormonal contraceptive use						
Never user	71 (29.8)	242 (25.4)	0.333	63 (37.7)	184 (27.5)	0.012
Ever user	162 (68.1)	681 (71.5)		96 (57.5)	464 (69.5)	
Missing	5 (2.1)	29 (3.0)		8 (4.8)	20 (3.0)	
Number of years used, median (IQR)	6 (2–10)	6 (3–12)		7 (3–10)	7 (2–13)	
Menopausal hormone therapy						
Never user	155 (65.1)	531 (55.8)	0.075	89 (53.3)	378 (56.6)	0.080
Previous user	65 (27.3)	316 (33.2)		49 (29.3)	221 (33.1)	
Current user	15 (6.3)	86 (9.0)		23 (13.8)	51 (7.6)	
Missing	3 (1.3)	19 (2.0)		6 (3.6)	18 (2.7)	
Number of years used, median (IQR)	4.5 (0–10)	5 (1–10)		6 (2–12)	5 (1–10)	
Charlson’s comorbidity index score						
0	218 (91.6)	895 (94.0)	0.217	154 (92.2)	608 (91.0)	0.945
1	9 (3.8)	33 (3.5)		8 (4.8)	34 (5.1)	
≥ 2	11 (4.6)	24 (2.5)		5 (3.0)	26 (3.9)	

*Col %* column percent; *ARIA* Accessibility Remoteness Index for Australia; *CCI* Charlson Comorbidity Index; *IQR* Interquartile range (25–75%)

*Numbers < 5 are suppressed to preserve confidentiality

^a^For controls, the age on the date of diagnosis of their matched case

^b^Characteristic used for matching controls to cases. Date of birth was also used for matching but is not shown here

^c^Some categories considered separately for matching of cases and controls are combined in this Table to preserve confidentiality where numbers are small

^d^Characteristic was derived using the reference method as described in Yap et al. [23]

### Healthcare utilization

**Fig. 1:**
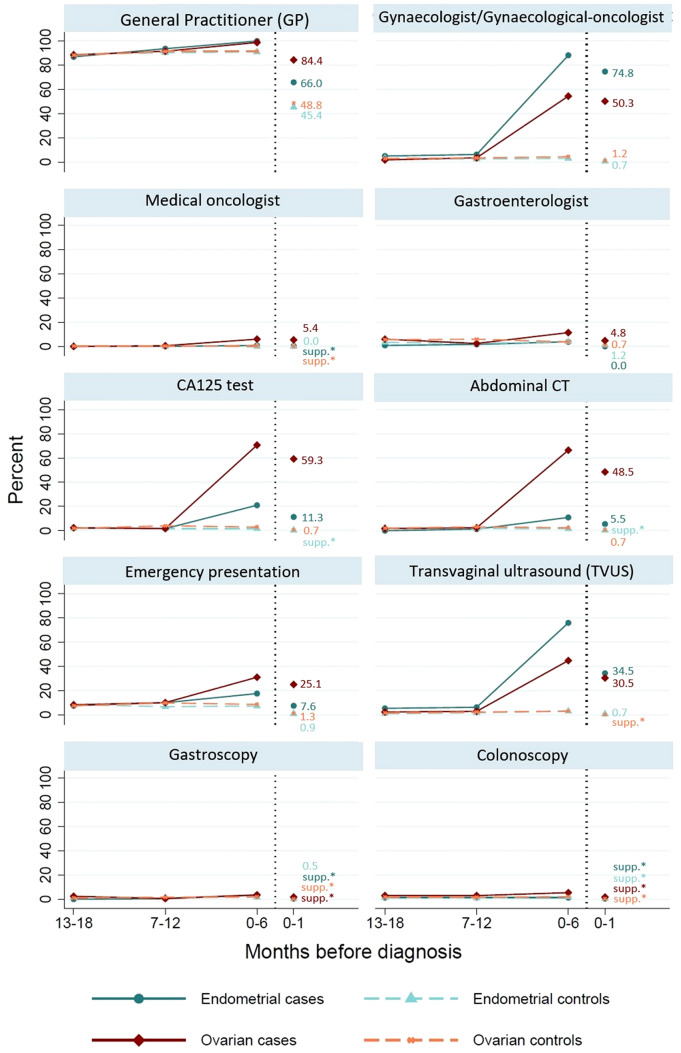
Percentage of ovarian and endometrial cancer cases and matched controls who received each health service at least once during each time interval prior to diagnosis. For each group of cases and controls, percentages representing small numbers (*n* < 5) are suppressed to preserve confidentiality (labeled as “supp.*”)

For endometrial cancer cases, healthcare utilization was generally similar to matched controls in the 13–18- and 7–12-months pre-diagnosis (differences ≤ 3.4%, Fig. 1), except for significantly higher proportions having records of TVUS during both periods (5.5% and 6.3%, respectively, versus < 2.0% of controls, *p* < 0.005; Supplementary Table 3) and of gynecologist/gynecological oncologist visits in the 7–12-months pre-diagnosis (6.3% versus 2.5% of controls, *p* = 0.007).

In the 0–6- and 0–1-months pre-diagnosis, significantly higher proportions of endometrial cancer cases than matched controls had records of gynecologist/gynecological oncologist visits (88.2% and 74.8% of cases, respectively), CA125 tests (21.0% and 11.3%), abdominal CT scans (10.9% and 5.5%), and TVUS (76.1% and 34.5%), compared to ≤ 3.0% of controls for each period and health service (Supplementary Table 3). Moreover, GP visits were significantly more common for endometrial cases than matched controls in the 0–6-months pre-diagnosis (100.0% versus 91.0%), as were emergency department visits (17.6% versus 7.4%). A post hoc analysis of each month in the 0–6-months pre-diagnosis showed healthcare utilization diverged for endometrial cancer cases and controls during the last 2-months pre-diagnosis (Supplementary Fig. 2), with only gynecologist/gynecological oncologist visits and TVUS beginning to diverge slightly earlier (4-months pre-diagnosis).

**Fig. 2:**
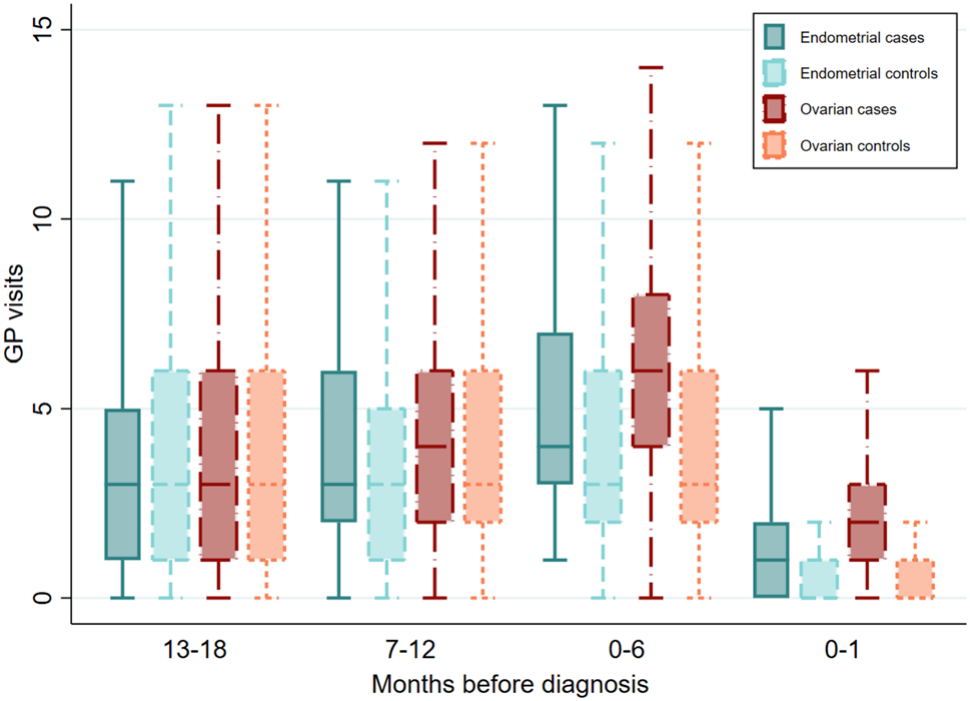
Number of GP visits for ovarian and endometrial cancer cases and matched controls during each time interval prior to diagnosis. The boxes show the 25–75% quantiles and the median (horizontal line), with whiskers extending to 1.5 times the interquartile range

In the 0–6-months pre-diagnosis, ~ 87% of the endometrial cancer cases had seen a gynecologist/gynecological oncologist only, very few had seen a medical oncologist only or both specialists, and ~ 11% did not have a record of visiting either of these specialists (excluding consultations for public in-patients throughout, see section “Methods”). Of the 28 endometrial cancer cases who did not have an MBS record for visits with either specialist, 18 (64.3%) had a hospital admission and/or emergency department visit in the 0–1-months pre-diagnosis. As at least 87.0% of cases and controls had GP consultation records in the 13–18-, 7–12-, and 0–6-months pre-diagnosis, we also considered the numbers of GP consultations per person (Fig. 2). Compared to their matched controls, endometrial cancer cases had similar numbers of GP consultations in the 13–18- and 7–12-months pre-diagnosis (both *p* > 0.5) and higher numbers in the 0–6- and 0–1-months pre-diagnosis (both *p* < 0.001).

For ovarian cancer cases, healthcare utilization was generally similar to matched controls in the 13–18- and 7–12-months pre-diagnosis (differences ≤ 1.4%, Fig. 1). For the 0–6-months and 0–1-months pre-diagnosis, significantly higher proportions of ovarian cancer cases had gynecologist/gynecological oncologist visits (54.5% and 50.3% of cases, respectively), CA125 tests (70.7% and 59.3%), abdominal CT scans (66.5% and 48.5%), and TVUS (44.9% and 30.5%) compared to ≤ 4.5% of matched controls for each period and health service (Supplementary Table 3). Additionally, in the 0–6-months pre-diagnosis, higher proportions of ovarian cases compared to controls had records of GP visits (98.8% versus 91.5%, respectively), medical oncologist visits (6.0% versus < 0.8%), gastroenterologist visits (11.4% versus 3.7%), and emergency department visits (31.1% versus 8.7%) (all *p* < 0.005). The post hoc analysis of each month in the 0–6-months pre-diagnosis demonstrated healthcare utilization for ovarian cancer cases and controls mostly diverged in the 1–2- or 0–1-months pre-diagnosis (Supplementary Fig. 2).

In the 0–6-months pre-diagnosis, 50.9% of ovarian cases had seen a gynecologist/gynecological oncologist, another 6% had seen a medical oncologist only or both specialists, while 43.1% did not have a record of visiting either of these specialists. Of the 72 ovarian cancer cases who did not have an MBS record for visits with either specialist, 57 (79.2%) had a hospital admission and/or emergency department visit in the 0–1-months pre-diagnosis.

In the analysis of the numbers of GP consultations per person (Fig. 2), ovarian cancer cases had similar numbers of GP consultations in the 13–18- and 7–12-months pre-diagnosis compared to their matched controls (both *p* > 0.5) and higher numbers in the 0–6- and 0–1-months pre-diagnosis (both *p* < 0.001).

### Sensitivity analysis

In a sensitivity analysis without exclusion of DVA clients, we compared healthcare utilization of 252 endometrial cancer cases (including 14 DVA clients) versus their 1,008 matched controls and of 181 ovarian cancer cases (including 14 DVA clients) versus their 724 matched controls. The results were highly similar to those from the main analysis (absolute change in estimated differences between healthcare utilization of cases and controls < 3.6%), and all statistically significant differences in the main analysis remained statistically significant. For example, in the 0–1-months pre-diagnosis, records of a gynecologist/gynecological oncologist visit were identified for 50.3% of ovarian cancer cases in the main analysis and 49.7% in the sensitivity analysis with differences to matched controls of 49.1% and 48.9%, respectively. Similarly, in the 0–1-months pre-diagnosis, records of emergency department visits were identified for 25.1% of ovarian cancer cases in the main analysis and 26.0% in the sensitivity analysis with differences to matched controls of 23.8% and 24.1%, respectively.

## Discussion

### Overview

We found increased healthcare utilization for women diagnosed with endometrial or ovarian cancer compared to matched controls in the 0–6 months and particularly the 0–1-months pre-diagnosis, including several types of clinical consultations, tests and procedures.

### Healthcare utilization of endometrial cancer cases

Recommendations for the diagnosis of endometrial cancer in Australia include a general and pelvic examination, referral for a TVUS, routine blood tests, and to a specialist gynecologist if TVUS is abnormal or there is clinical suspicion. The diagnosis is made based on endometrial sampling, either with office biopsy or hysteroscopy and dilatation and curettage (usually performed in hospital). After diagnosis, further imaging should be performed within 2 weeks of specialist review [5]. We found that, of women with endometrial cancer, 88.2% and 74.8% had seen a gynecologist/gynecological oncologist in the 0–6- and 0–1-months pre-diagnosis, respectively, aligning with the optimal care recommendations [5]. While a CA125 test is not explicitly recommended, 21.0% and 11.3% had a CA125 test in the same timeframes, and other studies indicate a CA125 test may assist the identification of extrauterine disease [28]. As recommended, 76.1% of endometrial cancer cases had a TVUS in the 0–6-months pre-diagnosis. While the overall uptake of TVUSs in the 7–12- and 13–18-months pre-diagnosis was small for both cases and controls, we found a slight increase in the use of these tests in these time periods (5.5–6.3% of cases versus 1.1–1.8% of controls). Similarly, 6.3% of women with endometrial cancer, versus 2.5% of controls, visited a gynecologist in the 7–12-months pre-diagnosis. This finding suggests that for a small proportion of cases, symptoms may have onset as early as 12-month pre-diagnosis and ‘watch and wait’ approaches may have been recommended [29]. Alternatively, these women may have been seeing a gynecologist and/or having investigations for other reasons associated with increased risk of endometrial cancer, such as Lynch syndrome [30]. Further investigations would be needed to dissect the pathway to diagnosis for these cases in more detail. There were no other differences between cases and controls for utilization of the other health services examined.

### Healthcare utilization of ovarian cancer cases

Recommendations for the diagnosis of ovarian cancer include a general and pelvic examination, referral for a TVUS and to a specialist gynecologist/gynecological oncologist within 2 weeks of a suspected diagnosis [5]. Additional preliminary tests for suspected ovarian cancer include calculation of the risk of malignancy index (a risk assessment tool [31]), CT scans, and routine blood tests, including CA125 [5, 32]. We found that 54.5% and 50.3% of ovarian cancer cases consulted a gynecologist/gynecological oncologist in the 0–6- and 0–1-months pre-diagnosis, respectively. Of the ovarian cancer cases who did not have a record of seeing either specialist pre-diagnosis, 79.2% were admitted to hospital in the 0–1-months pre-diagnosis, so may have consulted a specialist in that setting. For the recommended tests of CA125 and abdominal/pelvic CT scans, we saw similar patterns to those for specialist consultations, with a reasonably high uptake in the 6-months pre-diagnosis (70.7% and 66.5% of all ovarian cancer cases, respectively). Notably, fewer than half the women diagnosed with ovarian cancer had a TVUS in the 6-months pre-diagnosis. Given that 70% of women with ovarian cancer present with advanced intraabdominal disease, it may be that an abdominal CT is being used to investigate abdominal symptoms, rather than performing a TVUS.

In the 0–6-months pre-diagnosis, 11.4% of women diagnosed with ovarian cancer saw a gastroenterologist, compared to 3.7% of controls (with a post hoc analysis indicating that this difference was due to consultations in the 2-months pre-diagnosis), with no significant differences for colonoscopy or gastroscopy. This suggests that, while ruling out other explanations for abdominal symptoms is important, this largely occurs in the last months before diagnosis and thus does not greatly delay diagnosis. However, this finding could also be due to the exclusion of women aged < 45 years who may require more gastrointestinal investigations. For example, guidelines specify that irritable bowel syndrome rarely presents for the first time in women aged over 50 and thus ovarian cancer investigations should take priority [32].

There were no differences between ovarian cancer cases and controls for any of the consultations, tests or procedures, or for the number of GP visits, in the 7–12- or 13–18-months pre-diagnosis. Indeed, the post hoc analysis suggests that the differences in healthcare utilization only occur in the 2-months pre-diagnosis, suggesting that GPs are investigating and referring promptly. This aligns with previous Australian studies reporting that 80% of women were diagnosed within 3 months of their initial presentation [11] and other research emphasizing that tumor characteristics of ovarian cancer play a critical role in stage of diagnosis [7]. Once abdominal symptoms present, they are likely due to peritoneal spread [33], and therefore diagnosing ovarian cancer at an earlier stage may not be possible through improved health system processes alone [12], although efficient diagnostic processes certainly have benefits for an individual’s experience and a patient-centered approach to care [34]. However, as the recently published UKCTOCS study has shown, even when earlier diagnosis through screening results in a stage shift, there is no improvement in overall survival [35].

### Emergency department presentations

While the overall findings of this study provide evidence that the Australian health system is working to support timely diagnoses for the majority of women with endometrial or ovarian cancer, 25.1% of women diagnosed with ovarian cancer and 7.6% of women diagnosed with endometrial cancer visited an emergency department in the 0–1-months pre-diagnosis, significantly more than matched controls. Canadian and US studies have reported that emergency presentations are associated with poorer outcomes, including advanced disease at diagnosis and increased mortality [36, 37]. The reasons for emergency department visits are not captured within this study and would warrant future local research.

### Study strengths and limitations

While the findings of this study indicate several priorities for future research and action to support better outcomes for ovarian and endometrial cancers, this work has limitations. Despite the large size of the 45 and Up Study cohort, there were relatively small numbers of endometrial and ovarian cancer cases, so the findings should be interpreted with caution. The small numbers also precluded detailed analysis by stage, histology, or location of residence, which would provide valuable insights. Women aged < 45 years were not included in this analysis and likely have different diagnosis experiences due to the rarity of ovarian and endometrial cancers in this younger age group, as well as differences in ovarian disease histology. Data presented here are for women diagnosed up to 2013 only, so there may have been changes in practice since this time that are not captured in this study, for example, due to the publication of the second edition of the national Australian Optimal Care Pathways for endometrial and ovarian cancer in 2021 [5, 26]. Additionally, participants of the 45 and Up Study are generally better educated, healthier, and more health conscious than the general population, which could contribute to improved awareness of symptoms, seeking a diagnosis earlier or having a better relationship with their GP, all of which would impact the findings [38]. As the date of diagnosis in the NSWCR and thus this study represents the earliest definitive diagnostic or treatment episode, it may not reflect the exact date a clinician made the diagnosis. As the analysis was conducted using APDC, EDDC, and MBS records, information was not available on specialist consultations or tests conducted during hospital admissions for public patients in public hospitals, potentially underestimating the proportion of women who accessed recommended specialists and tests. Information was also not available regarding the reason for GP/specialist visits or emergency presentations nor individuals’ healthcare-related preferences and decisions. We also did not analyze the sequence of health system interactions, the sequence of test ordering, or which clinician ordered the tests. While the national Optimal Care Pathways recommend referral to a gynecological oncologist for definitive diagnosis and treatment, we were not able to separate gynecologist visits from gynecological oncologist visits in the available data. However, the case–control design of this study with close matching on age, smoking status, BMI, and place of residence allowed the identification of differences between women experiencing cancer and cancer-free women.

### Future actions

The findings of this study highlight some potential future directions for gynecological cancer patterns of care research and action. As access to specialist services has been shown to improve outcomes for ovarian cancer [39, 40], it would be valuable for future studies to further investigate referrals into specialist services and their access by place of residence or other sociodemographic characteristics that may be associated with different diagnostic experiences [41].

Our findings show that healthcare utilization diverges between women with endometrial or ovarian cancer and their matched controls primarily within the month pre-diagnosis. A large proportion of women see the recommended specialists and undertake the recommended tests and procedures, reinforcing the recommendations from the Optimal Care Pathways that timely referral to specialists and investigations is critical. However, a quarter of women with ovarian cancer visited emergency departments in the 0–1-months pre-diagnosis, warranting further research.

## Supplementary Information

Below is the link to the electronic supplementary material.

Supplementary file1 (DOCX 349 kb)

## Data Availability

The data used for this study were accessed using the Secure Unified Research Environment (SURE). The 45 and Up Study data and linked datasets are available from the Sax Institute, but restrictions apply to their availability. Therefore the data used for this study cannot be made publicly available. Researchers are able to access these data from the relevant data custodians for approved research projects, and enquiries for data access can be made to the Sax Institute (see https://www.saxinstitute.org.au/our-work/45-up-study/for-researchers/ for details).
