# Supplementary material for: Pathways to diagnosis of endometrial and ovarian cancer in the 45 and Up Study cohort
Source: Cancer Causes Control. 2022 Oct 9;34(1):47–58. doi: 10.1007/s10552-022-01634-2 (PMC9816254; doi:10.1007/s10552-022-01634-2)
Supplement: Supplementary file 1 — Supplementary file1 (DOCX 349 kb) [file 10552_2022_1634_MOESM1_ESM.docx]

**SUPPLEMENTARY TABLES AND FIGURES**

**Supplementary Table 1: Medicare Benefits Schedule item codes used to classify each health service**

| **Data type** | **Health service** | **Health service code** |
| --- | --- | --- |
| MBS item number | General Practitioner (GP) visit | 1, 2, 3, 4, 19, 20, 23, 24, 25, 33, 35, 36, 37, 40, 43, 44, 47, 48, 50, 51, 52, 53, 54, 57, 58, 59, 60, 65, 92, 93, 95, 96, 160, 161, 162, 163, 164, 597, 598, 599, 600, 701, 703, 705, 707, 715, 721, 723, 729, 731, 732, 735, 739, 743, 747, 750, 758, 871, 872, 900, 903, 5000, 5003, 5010, 5020, 5023, 5028, 5040, 5043, 5049, 5060, 5063, 5067, 5200, 5203, 5207, 5208, 5220, 5223, 5227, 5228, 5260, 5263, 5265, 5267 |
| MBS provider specialty number | Gynaecologist and Gynaecological Oncologist visit | For the year 2013^1^: 53, 54, 55 All other years (excluding 2013): 16, 75, 76 |
| MBS provider specialty number | Medical Oncologist visit | For the year 2013: 46 All other years (excluding 2013): 33 |
| MBS provider specialty number | Gastroenterologist visit | For the year 2013: 34 All other years (excluding 2013): 27 |
| MBS item number | CA125 test | 66650, 66651, 66652, 66653 |
| MBS item number | Gastroscopy | 30473, 30478, 30479, 41816, 41822 |
| MBS item number | Colonoscopy | 32090, 32084 |
| MBS item number | Abdominal Computed Tomography (CT) | 56401, 56407, 56441, 56447,56501, 56507, 56541, 56547, 56801, 56807, 65601 |
| MBS item number | Transvaginal ultrasound | 55065, 55067, 55068, 55731, 55733, 55735, 55736, 55737, 55739 |

^1^ Due to changes in MBS coding, different item numbers were used for 2013.

**Supplementary Table 2: Additional characteristics of endometrial and ovarian cancer cases and matched controls in the 45 and Up Study, New South Wales, Australia**

| **Characteristics** | **Endometrial cancer** | | | **Ovarian cancer** | | |
| --- | --- | --- | --- | --- | --- | --- |
|  | **Cases** | **Controls** | **Fishers exact test** | **Cases** | **Controls** | **Fishers exact test** |
|  | **N=238** | **N=952** |  | **N=167** | **N=668** |  |
|  | **n (col %)** | **n (col %)** | **p-value** | **n (col %)** | **n (col %)** | **p-value** |
| **Menopausal status (self-reported)** | |  |  |  |  |  |
| Pre-menopause | 11 (4.6) | 75 (7.9) | *<0.001* | 11 (6.6) | 57 (8.5) | *0.339* |
| Post-menopause | 189 (79.4) | 666 (70.0) |  | 134 (80.2) | 488 (73.1) |  |
| Peri-menopause | 24 (10.1) | 36 (3.8) |  | * | 24 (3.6) |  |
| Unknown | 14 (5.9) | 175 (18.4) |  | * | 99 (14.8) |  |
| **Family history of breast cancer** |  |  |  |  |  |  |
| Yes | 29 (12.2) | 111 (11.7) | *0.906* | 20 (12.0) | 66 (9.9) | *0.543* |
| No | 168 (70.6) | 685 (72.0) |  | 126 (75.4) | 529 (79.2) |  |
| Do not know or missing | 41 (17.2) | 156 (16.4) |  | 21 (12.6) | 73 (10.9) |  |
| **Family history of ovarian cancer** |  |  |  |  |  |  |
| Yes | 11 (4.6) | 30 (3.2) | *0.443* | 8 (4.8) | 18 (2.7) | *0.266* |
| No | 186 (78.2) | 767 (80.6) |  | 138 (82.6) | 577 (86.4) |  |
| Do not know or missing | 41 (17.2) | 155 (16.3) |  | 21 (12.6) | 73 (10.9) |  |
| **Family history of bowel cancer** |  |  |  |  |  |  |
| Yes | 21 (8.8) | 106 (11.1) | *0.598* | 27 (16.2) | 104 (15.6) | *0.787* |
| No | 176 (73.9) | 690 (72.5) |  | 119 (71.3) | 491 (73.5) |  |
| Do not know or missing | 41 (17.2) | 156 (16.4) |  | 21 (12.6) | 73 (10.9) |  |
| **K10 distress scale** |  |  |  |  |  |  |
| Well/Mild (0 to 24) | 196 (82.4) | 785 (82.5) | *0.298* | 128 (76.6) | 518 (77.5) | *0.347* |
| Moderate/Severe (25 to 50) | 6 (2.5) | 43 (4.5) |  | 6 (3.6) | 40 (6.0) |  |
| Missing | 36 (15.1) | 124 (13.0) |  | 33 (19.8) | 110 (16.5) |  |
| **Overall health** |  |  |  |  |  |  |
| Excellent/Very Good | 121 (50.8) | 479 (50.3) | *0.786* | 84 (50.3) | 318 (47.6) | *0.585* |
| Good | 81 (34.0) | 324 (34.0) |  | 56 (33.5) | 230 (34.4) |  |
| Fair/Poor | 31 (13.0) | 116 (12.2) |  | 24 (14.4) | 93 (13.9) |  |
| Missing | 5 (2.1) | 33 (3.5) |  | 3 (1.8) | 27 (4.0) |  |
| **Education** |  |  |  |  |  |  |
| University degree or higher | 48 (20.2) | 185 (19.4) | *0.145* | 30 (18.0) | 137 (20.5) | *0.508* |
| No university degree | 182 (76.5) | 753 (79.1) |  | 132 (79.0) | 518 (77.5) |  |
| Missing | 8 (3.4) | 14 (1.5) |  | 5 (3.0) | 13 (1.9) |  |
| **Married or de facto** |  |  |  |  |  |  |
| Yes | 171 (71.8) | 681 (71.5) | *1.000* | 111 (66.5) | 464 (69.5) | *0.456* |
| No | 67 (28.2) | 269 (28.3) |  | 56 (33.5) | 204 (30.5) |  |
| Missing |  | 2 (0.2) |  |  |  |  |

* Numbers <5 are suppressed to preserve confidentiality.

**Supplementary Table 3: Percentage of endometrial (n=238) and ovarian (n=167) cancer cases and matched controls (n=592 and n=952, respectively) with at least one record of the corresponding health service during each time interval prior to cancer diagnosis**

| **Healthcare service** | **Time interval prior to cancer diagnosis** | | | | | |
| --- | --- | --- | --- | --- | --- | --- |
|  | **Including day of diagnosis** | | | | **Excluding day of diagnosis** | |
|  | **13-18 months** | **7-12 months** | **0-6 months** | **0-1 months** | **0-6 months** | **0-1 months** |
| **GP** |  |  |  |  |  |  |
| Endometrial cases | 87.0 | 93.7 | 100.0 | 66.0 | 100.0 | 64.7 |
| Endometrial controls | 88.9 | 90.3 | 91.0 | 45.4 | 91.1 | 44.9 |
| *p-value* | *0.427* | *0.128* | *<0.005^* | *<0.005^* | *<0.005^* | *<0.005^* |
| Ovarian cases | 88.6 | 91.6 | 98.8 | 84.4 | 98.8 | 83.8 |
| Ovarian controls | 88.3 | 91.5 | 91.5 | 48.8 | 91.5 | 47.9 |
| *p-value* | *1.000* | *1.000* | *<0.005^* | *<0.005^* | *<0.005^* | *<0.005^* |
| **Gynaecologist/Gynaecological-oncologist** | | |  |  |  |  |
| Endometrial cases | 5.0 | 6.3 | 88.2 | 74.8 | 82.8 | 59.2 |
| Endometrial controls | 2.9 | 2.5 | 3.0 | 0.7 | 3.0 | 0.7 |
| *p-value* | *0.110* | *0.007* | *<0.005^* | *<0.005^* | *<0.005^* | *<0.005^* |
| Ovarian cases | <3.0* | 3.6 | 54.5 | 50.3 | 50.3 | 44.9 |
| Ovarian controls | 3.0 | 3.4 | 4.5 | 1.2 | 4.3 | 1.0 |
| *p-value* | *0.597* | *1.000* | *<0.005^* | *<0.005^* | *<0.005^* | *<0.005^* |
| **Medical oncologist** |  |  |  |  |  |  |
| Endometrial cases | 0.0 | 0.0 | <2.1* | <2.1* | <2.1* | <2.1* |
| Endometrial controls | <0.5* | <0.5* | 0.0 | 0.0 | 0.0 | 0.0 |
| *p-value* | *1.000* | *1.000* | *0.040* | *0.200* | *0.040* | *0.200* |
| Ovarian cases | 0.0 | <3.0* | 6.0 | 5.4 | 4.2 | 3.6 |
| Ovarian controls | <0.8* | <0.8* | <0.8* | <0.8* | <0.8* | <0.8* |
| *p-value* | *1.000* | *0.489* | *<0.005^* | *<0.005^* | *<0.005^* | *<0.005^* |
| **Gastroenterologist** |  |  |  |  |  |  |
| Endometrial cases | <2.1* | <2.1* | 3.8 | 0.0 | <2.1* | 0.0 |
| Endometrial controls | 3.4 | 2.5 | 3.9 | 1.2 | 3.9 | 1.1 |
| *p-value* | *0.047* | *0.632* | *1.000* | *0.135* | *1.000* | *0.226* |
| Ovarian cases | 6.0 | <3.0* | 11.4 | 4.8 | 10.8 | 4.8 |
| Ovarian controls | 5.5 | 6.0 | 3.7 | <0.8* | 3.7 | <0.8* |
| *p-value* | *0.851* | *0.079* | *<0.005^* | *<0.005^* | *<0.005^* | *<0.005^* |
| **CA125 test** |  |  |  |  |  |  |
| Endometrial cases | 2.1 | <2.1* | 21.0 | 11.3 | 19.3 | 9.7 |
| Endometrial controls | 2.1 | 1.7 | 1.7 | <0.5* | 1.7 | <0.5* |
| *p-value* | *1.000* | *1.000* | *<0.005^* | *<0.005^* | *<0.005^* | *<0.005^* |
| Ovarian cases | <3.0* | <3.0* | 70.7 | 59.3 | 66.5 | 55.1 |
| Ovarian controls | 1.9 | 4.0 | 3.0 | <0.8* | 3.0 | <0.8* |
| *p-value* | *0.759* | *0.243* | *<0.005^* | *<0.005^* | *<0.005^* | *<0.005^* |
| **Abdominal/pelvic CT scan** |  |  |  |  |  |  |
| Endometrial cases | 0.0 | <2.1* | 10.9 | 5.5 | 10.5 | 5.0 |
| Endometrial controls | 2.0 | 1.8 | 1.6 | <0.5* | 1.6 | <0.5* |
| *p-value* | *0.020* | *0.780* | *<0.005^* | *<0.005^* | *<0.005^* | *<0.005^* |
| Ovarian cases | <3.0* | <3.0* | 66.5 | 48.5 | 62.9 | 46.1 |
| Ovarian controls | 2.1 | 3.1 | 2.4 | <0.8* | 2.4 | <0.8* |
| *p-value* | *1.000* | *0.801* | *<0.005^* | *<0.005^* | *<0.005^* | *<0.005^* |
| **Emergency department visit** | |  |  |  |  |  |
| Endometrial cases | 7.6 | 10.1 | 17.6 | 7.6 | 17.6 | 7.6 |
| Endometrial controls | 8.4 | 6.8 | 7.4 | 0.9 | 7.4 | 0.9 |
| *p-value* | *0.792* | *0.098* | *<0.005^* | *<0.005^* | *<0.005^* | *<0.005^* |
| Ovarian cases | 8.4 | 10.2 | 31.1 | 25.1 | 21.6 | 13.2 |
| Ovarian controls | 7.6 | 9.9 | 8.7 | 1.3 | 8.7 | 1.5 |
| *p-value* | *0.747* | *0.886* | *<0.005^* | *<0.005^* | *<0.005^* | *<0.005^* |
| **Transvaginal ultrasound (TVUS)** | |  |  |  |  |  |
| Endometrial cases | 5.5 | 6.3 | 76.1 | 34.5 | 75.6 | 34.5 |
| Endometrial controls | 1.1 | 1.8 | 3.0 | 0.7 | 3.0 | 0.7 |
| *p-value* | *<0.005^* | *<0.005^* | *<0.005^* | *<0.005^* | *<0.005^* | *<0.005^* |
| Ovarian cases | <3.0* | 3.0 | 44.9 | 30.5 | 44.3 | 29.9 |
| Ovarian controls | 1.6 | 2.4 | 3.1 | <0.8* | 3.1 | <0.8* |
| *p-value* | *0.516* | *0.589* | *<0.005^* | *<0.005^* | *<0.005^* | *<0.005^* |
| **Gastroscopy** |  |  |  |  |  |  |
| Endometrial cases | 0.0 | <2.1* | 3.4 | <2.1* | 3.4 | <2.1* |
| Endometrial controls | 1.5 | 1.3 | 1.7 | 0.5 | 1.6 | <0.5* |
| *p-value* | *0.086* | *0.484* | *0.119* | *1.000* | *0.108* | *1.000* |
| Ovarian cases | <3.0* | <3.0* | 3.6 | <3.0* | 3.6 | <3.0* |
| Ovarian controls | 1.0 | 1.6 | 1.9 | <0.8* | 1.9 | <0.8* |
| *p-value* | *0.244* | *0.477* | *0.241* | *0.027* | *0.241* | *0.027* |
| **Colonoscopy** |  |  |  |  |  |  |
| Endometrial cases | <2.1* | <2.1* | <2.1* | <2.1* | <2.1* | <2.1* |
| Endometrial controls | 1.5 | 1.1 | 2.0 | <0.5* | 2.0 | <0.5* |
| *p-value* | *1.000* | *0.731* | *0.596* | *1.000* | *0.596* | *1.000* |
| Ovarian cases | 3.0 | 3.0 | 5.4 | <3.0* | 5.4 | <3.0* |
| Ovarian controls | 1.8 | 1.6 | 2.1 | <0.8* | 2.1 | <0.8* |
| *p-value* | *0.356* | *0.338* | *0.031* | *0.057* | *0.031* | *0.057* |

* Percentages representing small numbers (n<5) are suppressed to preserve confidentiality.

^ Significant after Bonferroni correction for 10 health service types tested (i.e, p<0.05/10).

^1^ P-values are calculated from Fisher’s exact test.

**Excluded (N=114,525)**

**- NSWCR record (n=6,640)**

**- Missing matching variables (n=12,672)**

**- Controls not required (n=95,213)**

**Controls for analysis (N=1,620)**

**- Endometrial (n=952)**

**- Ovarian (n=668)**

**Excluded (N=77)**

**- Prior NSWCR record (n=51)**

**- Missing matching variables (n=26)**

**Excluded (N=52)**

**- Prior NSWCR record (n=33)**

**- Unspecified or non-epithelial histology (n=13)**

**- Missing matching variables (n=6)**

**Endometrial cases for analysis (n=238)**

**Ovarian cases for analysis (n=167)**

**Endometrial cancer recorded after baseline (n=315)**

**Ovarian cancer recorded after baseline (n=219)**

**Potential controls (n=116,145)**

**45 and Up Study female participants (n=142,973)**

**Excluded (N=26,324)**

**- Linkage error (n=15)**

**- DVA client (n=6,401)**

**- Self-reported cancer except NMSC (n=19,908)**

**Participants linked to NSWCR** **(n=116,649)**

**Supplementary Figure 1: Flowchart illustrating the selection of endometrial and ovarian cancer cases and matched controls from the 45 and Up Study cohort.** Each case was matched to four female controls based on date of birth, tobacco smoking, body mass index and place of residence.
DVA: Department of Veterans’ Affairs; NMSC: Non-melanoma skin cancers; NSWCR: New South Wales Cancer Registry


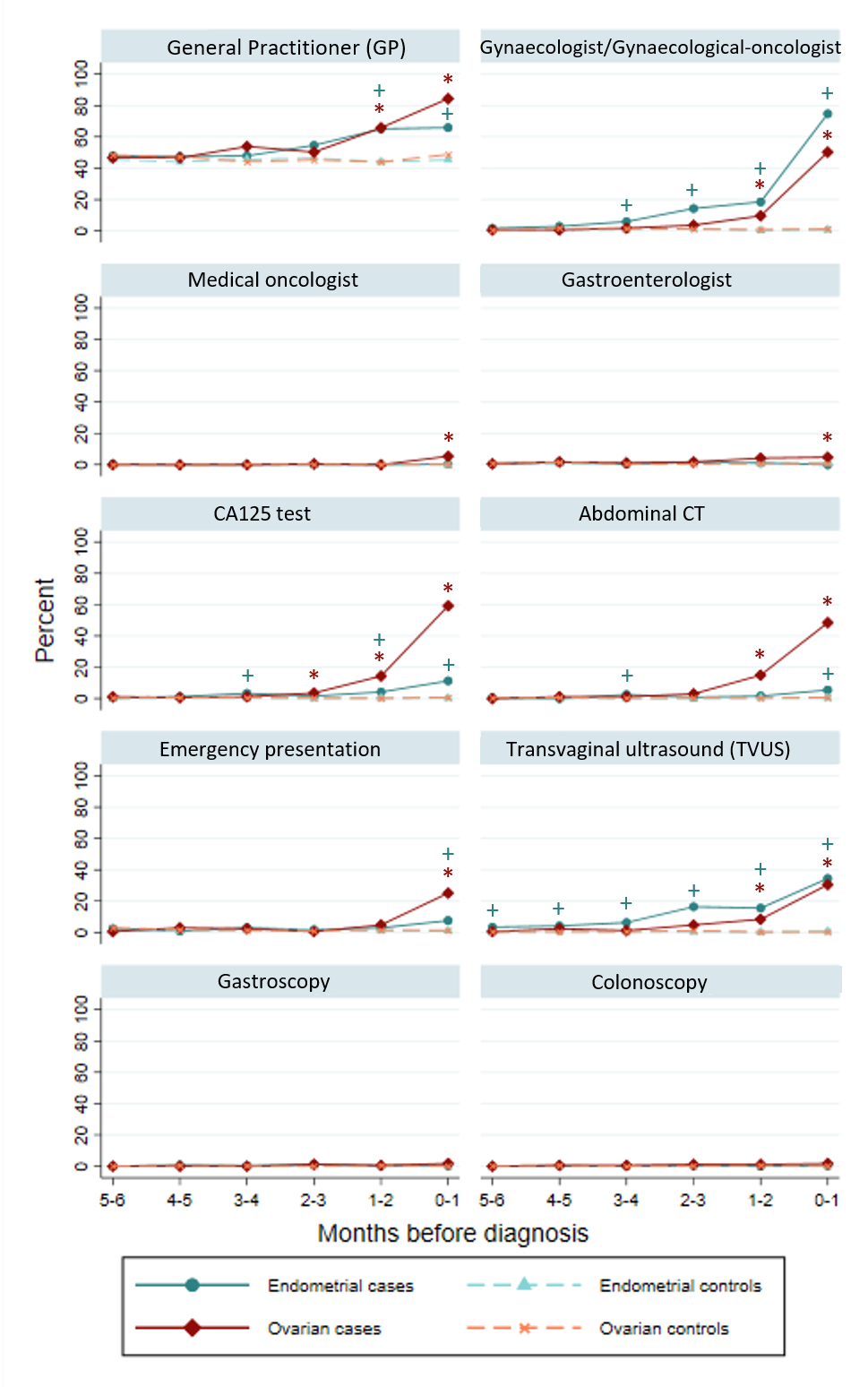


**Supplementary Figure 2: Percentage of endometrial and ovarian cancer cases and matched controls who received the health service at least once during each time interval prior to diagnosis.** For each time interval, significant differences after Bonferroni correction for 10 health service types (i.e, p<0.005) are indicated by “+” for differences between endometrial cancers cases and matched controls, and by “*” for differences between ovarian cancer cases and matched controls.
